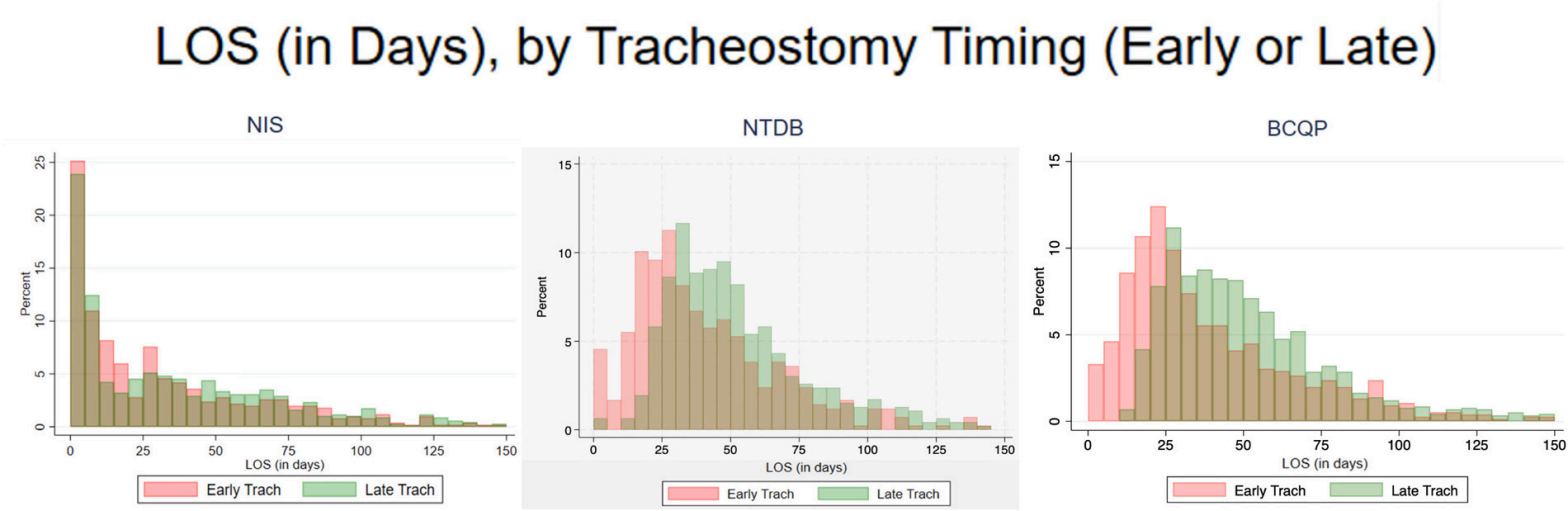# 52 Early versus Late Tracheostomy in Critically Injured Burn Patients

**DOI:** 10.1093/jbcr/iraf019.052

**Published:** 2025-04-01

**Authors:** Jennifer Shah, Eloise Stanton, Daniel Najafali, Clifford Sheckter

**Affiliations:** Geisel School of Medicine at Dartmouth; University of Southern California; Carle Illinois College of Medicine; Santa Clara Valley Medical Center

## Abstract

**Introduction:**

Tracheostomy is indicated in critically injured burn patients when prolonged mechanical ventilation is anticipated. Meta-analyses in non-burn patients have shown early tracheostomy is associated with fewer ventilator days and shorter length of stay (LOS). Limited single institution studies have evaluated early tracheostomy in burn patients and found no benefits. We aimed to use national data to reevaluate the timing of tracheostomy in critically injured burn patients, hypothesizing that early tracheostomy would be associated with reduced LOS and reduced ventilator-associated pneumonia (VAP).

**Methods:**

Burn encounters undergoing tracheostomy were identified in three separate national databases—the Nationwide Inpatient Sample (NIS), 2012–2021, the US National Trauma Data Bank (NTDB), 2007–2018, and the Burn Care Quality Platform (BCQP), 2013-2022. Encounters were stratified by tracheostomy timing with respect to admission: early: ≤ 10 days vs. late: > 10 days. Propensity score matching was performed to adjust for confounding by indication utilizing 1:1 nearest-neighbor matching without replacement with maximum caliper distance of 0.1. Early tracheostomy patients were matched with late tracheostomy patients on age, sex, and total body surface area (TBSA) burned. Outcomes included overall LOS, ICU LOS, ventilator days, and VAP. Only burn survivors were included.

**Results:**

Tracheostomy was performed in 10,455 encounters in NIS, 1,852 encounters in NTDB, and 1,599 patients in BCQP. The median day for tracheostomy in NIS was 12.0 (IQR, 6.0, 18.0), 10.4 in NTDB (IQR, 5.7, 16.5), and 13.0 (IQR 8.0, 19.0) in BCQP. After matching, standardized mean differences (SMDs) were ≤ 0.1. Within this matched cohort, early tracheostomy was associated with shorter LOS in all databases, reported as Average Treatment Effect: NIS -5.7 days (95% CI: -10.7– -0.7; p=0.03), NTDB -11.1 days (95% CI: -16.3– -6.0; p< 0.01), BCQP -9.3 days (95% CI: -13.9– -4.7; p< 0.01). Early tracheostomy was additionally associated with shorter ICU LOS in NTDB (-7.7 days; 95% CI: -12.2– -3.3; p< 0.01) and BCQP (-6.2 days; 95% CI: -10.5– -2.0; p< 0.01). In the BCQP, early tracheostomy showed reduced ventilator days by -5.4 days (95% CI: -9.5– -1.2; p=0.01) Early tracheostomy was not associated with reduced VAP in any database.

**Conclusions:**

In a multi-database analysis with almost 14,000 encounters, early tracheostomy (within 10 days of admission) was associated with decreased ICU LOS, overall LOS, and fewer ventilator days, but did not affect VAP.

**Applicability of Research to Practice:**

For patients with anticipated prolonged mechanical ventilation, burn practitioners should consider tracheostomy within 10 days of admission.

**Funding for the Study:**

N/A